# Establishment of disulfidptosis-related LncRNA signature as biomarkers in colon adenocarcinoma

**DOI:** 10.1186/s12935-024-03374-6

**Published:** 2024-05-27

**Authors:** Hongfei Yao, Peng Liu, Linli Yao, Xiao Li

**Affiliations:** 1 Shanghai Sixth People’s Hospital Affiliated to Shanghai Jiao Tong University School of Medicine, Shanghai, 200233 People’s Republic of China; 2 State Key Laboratory of Oncogenes and Related Genes, Department of Biliary-Pancreatic Surgery, Ren Ji Hospital, Shanghai Jiao Tong University School of Medicine, Shanghai, 200127 People’s Republic of China; 3 State Key Laboratory of Oncogenes and Related Genes, Shanghai Cancer Institute, Ren Ji Hospital, School of Medicine, Shanghai Jiao Tong University, 800 Dongchuan Road, Shanghai, 200240 People’s Republic of China; 4 Department of Radiotherapy, The Affiliated Yantai Yuhuangding Hospital of Qingdao University, Yantai, 264000 China

**Keywords:** COAD, Disulfidptosis, Risk signature, lncRNAs

## Abstract

**Purpose:**

Metabolic reprogramming is a hallmark of cancer and plays a key role in precision oncology treatment. Long non-coding RNAs (lncRNAs) regulate cancer cell behavior, including metabolism. Disulfidptosis, a newly identified form of regulated cell death triggered by glucose starvation, has yet to be fully understood in colon adenocarcinoma (COAD). This study aimed to confirm the existence and role of disulfidptosis in COAD and identify disulfidptosis-related lncRNAs that may be targeted to induce disulfidptosis in COAD.

**Methods:**

PI and F-actin staining were used to observe disulfidptosis in COAD cell lines. Disulfidptosis-related lncRNAs were identified based on the expression of disulfidptosis-associated genes in the TCGA-COAD database. A four-lncRNA signature for disulfidptosis was established. Subsequently, loss-of-function assays explored the roles of AC013652.1 and MCM3AP-AS1 in disulfidptosis.

**Results:**

Disulfidptosis was observed in COAD cells under glucose starvation and could be reversed by agents that prevent disulfide stress, such as dithiothreitol (DTT) and tris-(2-carboxyethyl)-phosphine (TCEP). The prognostic value of disulfidptosis-associated genes in COAD patients was confirmed, with higher expression indicating longer survival. A disulfidptosis-related lncRNA signature comprising four lncRNAs was established based on the expression of these genes. Among these, AC013652.1 and MCM3AP-AS1 predicted worse prognoses. Furthermore, inhibiting AC013652.1 or MCM3AP-AS1 increased disulfidptosis-associated gene expression and cellular death, which could be reversed by DTT and TCEP.

**Conclusions:**

This study provides hitherto undocumented evidence of the existence of disulfidptosis and the prognostic value of disulfidptosis-associated genes in COAD. Importantly, we identified lncRNAs AC013652.1 and MCM3AP-AS1, which suppress disulfidptosis and may serve as potential therapeutic targets for COAD.

**Supplementary Information:**

The online version contains supplementary material available at 10.1186/s12935-024-03374-6.

## Introduction

Regulated cell death (RCD) is a universal, controlled form of cell death found in living organisms. It plays an important role in both physiological and pathological processes by maintaining tissue homeostasis and restoring biological balance under stress conditions [1–3]. Disulfidptosis, a newly identified metabolism-related RCD, was initially reported by Liu et al. [4]. This process describes how cells can program their death in response to a cystine imbalance under glucose starvation by inducing extensive disulfide bonding within the actin cytoskeleton. Carcinogenesis is a multifactorial and multistage process that requires sufficient metabolic resources [5–7]. It is well-known that cancer cells encounter various metabolic stresses during growth and metastasis. Therefore, metabolic reprogramming has been recognized as one of the hallmarks of cancer [8–10]. As a result, targeting cancer metabolism to selectively kill cancer cells has become a widely adopted strategy in the era of precision oncology [11]. Given disulfidptosis’s role as a metabolism-related RCD, investigating its role in cancer development is warranted.

Colorectal cancer (CRC) is one of the most common and deadliest human malignancies. It ranks as the third most common cancer and the second leading cause of cancer-related deaths worldwide [12–14]. Colon adenocarcinoma (COAD), the most prevalent type of CRC [15], presents a significant challenge with an unfavorable curative effect and poor prognosis. Approximately 2 million new cases and 600,000 deaths occur annually, with late diagnosis being a major contributor to the poor prognosis [16]. Epidemiological studies have identified numerous risk factors for colorectal cancer, including age, gender, inflammatory bowel disease, and lifestyle or environmental changes. These factors are particularly concerning in developed countries, where the incidence rate of CRC is on the rise. While total mesorectal excision remains the standard surgical approach for COAD, radical surgery and subsequent chemotherapy come with severe side effects. Despite the unprecedented medical progress achieved, more than half of patients diagnosed with COAD do not survive beyond five years. The lack of effective drugs for COAD treatment in clinical practice highlights the need for novel targeted therapies. Integrating such agents into standard therapy regimens holds the potential to improve patient survival and quality of life. This requires a deeper understanding of colon cancer biology and the identification of new druggable targets.

Long non-coding RNAs (lncRNAs), regulatory RNA molecules with limited or no protein-coding capacity, play a role in tumorigenesis and progression [17–19]. They exert extensive regulatory effects on various biological processes, including cell metabolism and RCD [20–23]. Studies have revealed the regulation of ferroptosis or pyroptosis by lncRNAs [24]. For instance, ferroptosis-related lncRNAs have been identified to predict the clinical outcome and molecular characteristics of pancreatic ductal adenocarcinoma. However, the regulation of disulfidptosis, a newly discovered metabolism-related RCD, by lncRNA remains unexplored.

This study assessed the differentially expressed disulfidptosis-associated genes and identified, for the first time, the existence of disulfidptosis under starvation conditions in colon adenocarcinoma. We then established a prognostic model based on disulfidptosis-related lncRNAs and utilized multiple bioinformatics approaches to explore the correlation between the risk model and prognosis, clinicopathology, and the tumor immune microenvironment in COAD. Therefore, identifying disulfidptosis-related lncRNAs is significant for deciphering the underlying mechanism of COAD tumourigenesis and exploring new disulfidptosis-targeted treatments.

## Materials and methods

### Data collection and arrangement

RNA-sequencing (RNA-seq) data was acquired from The Cancer Genome Atlas (TCGA: https://www.cancer.gov/ccg/research/genome-sequencing/tcga). This dataset included 448 colon cancer samples and 41 normal samples along with corresponding clinical information. Samples without clinical data were then excluded.

### Identification of disulfidptosis-related lncRNAs

Genes involved in disulfidptosis were acquired from prior research. Disulfidptosis-related lncRNAs (DRLs) were then identified using Pearson correlation analysis with a threshold of |Pearson R|> 0.4 and p-value < 0.001 [25]. Finally, a total of 100 DRLs were selected for further analysis.

### Establishment of the disulfidptosis-related risk signature

One hundred COAD samples were randomly separated into two equal cohorts for training and testing purposes. The DRLs were then filtered by univariate Cox regression analysis in the training cohort to identify potential prognostic DRLs (p < 0.05). Subsequently, least absolute shrinkage and selection operator (LASSO) and multivariate Cox regression analysis were applied to these DRLs to construct the disulfidptosis-related risk signature. The risk score of every COAD sample was calculated using the following formula:

$$\text{Risk score }=\Sigma [\text{Exp }(\text{lncRNA}) \times \text{ coef }(\text{lncRNA})]$$

The Exp (lncRNA) and coef (lncRNA) represent the expression level of each DRL and its corresponding coefficient in the risk score formula. Based on the median risk score, the training cohort samples were divided into low- and high-risk groups. The ‘survmine’ and ‘survival’ R packages were then used to generate a Kaplan–Meier curve with a log-rank test for survival analysis. Additionally, a receiver operating characteristic curve (ROC) was constructed using the ‘timeROC’ R package to evaluate the model’s performance. Finally, the risk signature’s validity was assessed in both the testing cohort and the entire cohort.

### Identify the different clusters of COAD samples based on disulfidptosis associated genes

To investigate the expression patterns of disulfidptosis associated genes in COAD samples, the ssGSEA was conducted. The disulfidptosis-score of every COAD sample was calculated based on the expression levels of disulfidptosis associated genes, and samples were separated into different clusters. ‘GSVA’, and ‘GSEABase’ R packages were utilized in the section. After that, the differentially expressed genes (DEGs) between different clusters were identified by the ‘limma’ R package with a criterion of |log2(foldchange)|> 1 and adjust p < 0.05.

### Functional analysis and gene set enrichment analysis (GSEA)

To explore the functional differences between the two groups, DEGs were identified with a threshold of |log2(foldchange)|> 1 and adjusted p-value < 0.05 using the ‘limma’ R package. Gene Ontology (GO) and Kyoto Encyclopedia of Genes and Genomes (KEGG) enrichment analysis were then conducted using the ‘clusterProfiler’, ‘enrichplot’, and ‘org.Hs.e.g.db’ packages [26] to identify significantly enriched pathways. Additionally, GSEA was performed using MSigDB’s GO and KEGG gene sets to further explore functional differences between the groups.

### Nomogram and calibration curves

To establish a reliable method for predicting the prognosis of COAD patients, a nomogram was constructed by integrating the risk score with other clinical characteristics. The nomogram allowed for the calculation of 1-, 2-, and 3-year survival probabilities. The calibration curve was then used to assess the accuracy of these predictions.

### Analysis of tumor immune microenvironment and the risk signature

Several algorithms were employed to explore the correlation between the risk signature and the tumor immune microenvironment. The CIBERSORT algorithm [27] was used to estimate the infiltration level of various immune cell types within each sample. Additionally, ssGSEA was performed using the ‘GSEAbase’ R package [28] to evaluate immune-related functions. Finally, Pearson correlation analysis was conducted to assess the correlation between the risk score and the abundance of specific immune cell populations.

### Culture of human colon cancer cells

The colon cancer cell lines HCT116, LoVo, HT29, and DLD-1 were purchased from the Cell Bank of the Chinese Academy of Sciences (Shanghai, China). These cells were cultured in McCoy’s 5A medium supplemented with 10% fetal bovine serum (FBS) and 1% penicillin/streptomycin (P/S). All cultures were maintained in a humidified incubator at 37 °C and 5% CO_2_.

### RNA extraction and quantitative real-time PCR (qRT-PCR)

Total RNA was extracted from cells using TRIzol reagent (Takara Bio, Dalian, China) and then reverse-transcribed into cDNA using the PrimeScript RT Master Mix reagent (Takara Bio, Dalian, China). The 2^−ΔΔCT^ method was employed to calculate the expression level of each gene, with GAPDH serving as the internal reference gene. Primer sequences for this study are provided in Supplementary Table 3.

### Cell transfection

For the gene knockdown assay, colon cancer cells were seeded into six-well plates at a desired density. After 24 h, transfection was performed using siRNA and Lipofectamine 2000 transfection reagent (Thermo Fisher, USA) according to the manufacturer’s instructions. Following 48 h of transfection, cells were harvested for further analysis. Knockdown efficiency was then assessed by qRT-PCR.

### Fluorescent staining of actin filaments

Cells were fixed with 4% paraformaldehyde, permeabilized with 0.5% Triton X-100 for 5 min at room temperature, and then washed with PBS. Subsequently, cells were stained with 100 nM Actin-stain 555 phalloidin (Thermo Fisher, USA) for 30 min at room temperature, followed by washing with PBS and staining with DAPI (Thermo Fisher, USA) for 5 min. All images were captured using a confocal microscope (LSM 880, Zeiss).

### Cell death assay

Cells were seeded into six-well plates and cultured in medium with or without glucose and appropriate drugs. Afterward, the cells were collected for analysis of dead (PI-positive) cells by FACS using BD AccuriTM C6 Plus cell analyzers (BD Biosciences, USA). The results were then analyzed using FlowJo 10.4 software.

### Statistical analysis

Bioinformatic analyses were performed using R software (version 4.0.2). Numerical data were analyzed using GraphPad Prism 8.4.3. Student’s t-test or two-way ANOVA were applied for comparisons between groups, as appropriate. The log-rank test was used to compare survival times in Kaplan–Meier survival curves. The Mann–Whitney test was used to compare ssGSEA scores. All statistical tests were considered significant at p < 0.05.

## Results

### Identification of disulfidptosis and the prognostic value of disulfidptosis associated genes in COAD

**Fig. 1:**
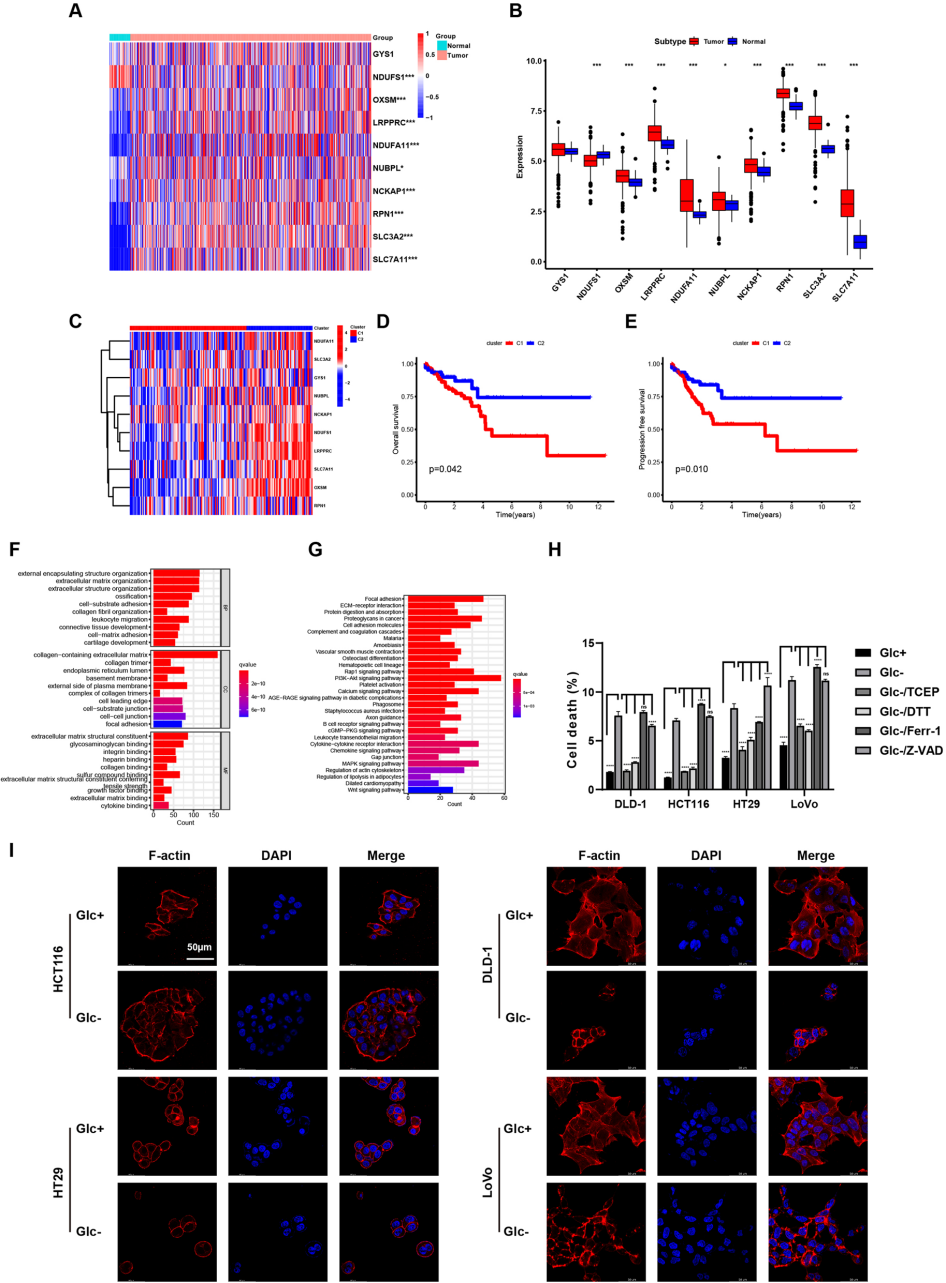
Disulfidptosis suppressed COAD development. **A** and **B** Heatmap (**A**) and graph (**B**) showing the mRNA expression of disulfidptosis associated genes in COAD tumor tissues and the normal control tissue. **C**–**E** The Cancer Genome Atlas (TCGA) RNA sequencing data of COAD were divided into two groups based on expression of disulfidptosis -related genes (**C**). Kaplan–Meier curve of OS (**D**) and PFS (**E**) between the two groups. **F** and **G** GO (**F**) and KEGG (**G**) enrichment analysis of increased expressed genes in disulfidptosis -related genes low-expressing group compared with high-expressing group. **H** Graph showing the PI positive dead cells after cultured in glucose-free medium for 12 h and that treated with 0.5 mM dithiothreitol (DTT), 1 mM tris-(2-carboxyethyl)-phosphine (TCEP), 10 μM Ferrostatin-1 (Ferr-1), and 10 μm Z-VAD-FMK (Z-VAD) in human COAD cell lines as analyzed by flowcytometry analysis, the statistical analyses were done by two-way ANOVA. **I** Fluorescence staining of F-actin with phalloidin in COAD cells after glucose starvation for the 12 h. Scale bar = 50 μm. ****p < 0.0001; ***p < 0.001; **p < 0.01; *p < 0.05

We first investigated the expression and prognostic value of disulfidptosis-associated genes in colon adenocarcinoma using the TCGA-COAD cohort. Ten genes reported to be associated with disulfidptosis were identified based on published literature (Supplementary Table 1). We analyzed the mRNA levels of these genes and found that eight out of the ten genes were significantly upregulated in primary COAD compared to normal colon tissue (Fig. 1A, B). Additionally, patients with high expression of the disulfidptosis-associated genes exhibited significantly longer overall survival (OS) and progression-free survival (PFS) compared to those with low expression (Fig. 1C–E). Consistent with these findings, GO and KEGG enrichment analysis revealed that multiple pathways promoting tumor progression were enriched in the low-expression group of disulfidptosis-associated genes compared to the high-expression group (Fig. 1F, G).

We next aimed to experimentally verify the existence of disulfidptosis in COAD cells under glucose starvation conditions. Four colon cancer cell lines, HCT116, HT29, DLD-1, and LoVo, were selected for this functional study. Firstly, we detected the expression level of disulfidptosis associated genes in DLD-1 and HCT116 under glucose starvation. The results showed that the most disulfidptosis associated genes was upregulated except SLC7A11 and SLC3A2, which confirmed the involvement of disulfidptosis in colon cancer (Supplementary Fig. 1A-B). The percentage of PI positive death cells were significantly increased after glucose starvation for 12 h, and the cell death was obviously rescued by dithiothreitol (DTT) and tris-(2-carboxyethyl)-phosphine (TCEP), which can prevent disulfide stress and suppress disulfidptosis. However, the death cells could not be rescued by ferroptosis inhibitors ferrostatin-1 (Ferr-1) and apoptosis inhibitor Z-VAD-FMK (Z-VAD) (Fig. 1H, Supplementary Fig. 1C). Furthermore, fluorescence staining revealed striking changes in cell morphology. Compared to cells cultured in glucose-containing medium, cells under glucose starvation exhibited cell shrinkage and F-actin contraction (Fig. 1I). These findings support the prognostic value of disulfidptosis-associated genes and suggest the involvement of disulfidptosis in COAD progression.

### Construction of a disulfidptosis‑related lncRNA predictive signature in colon adenocarcinoma

**Fig. 2:**
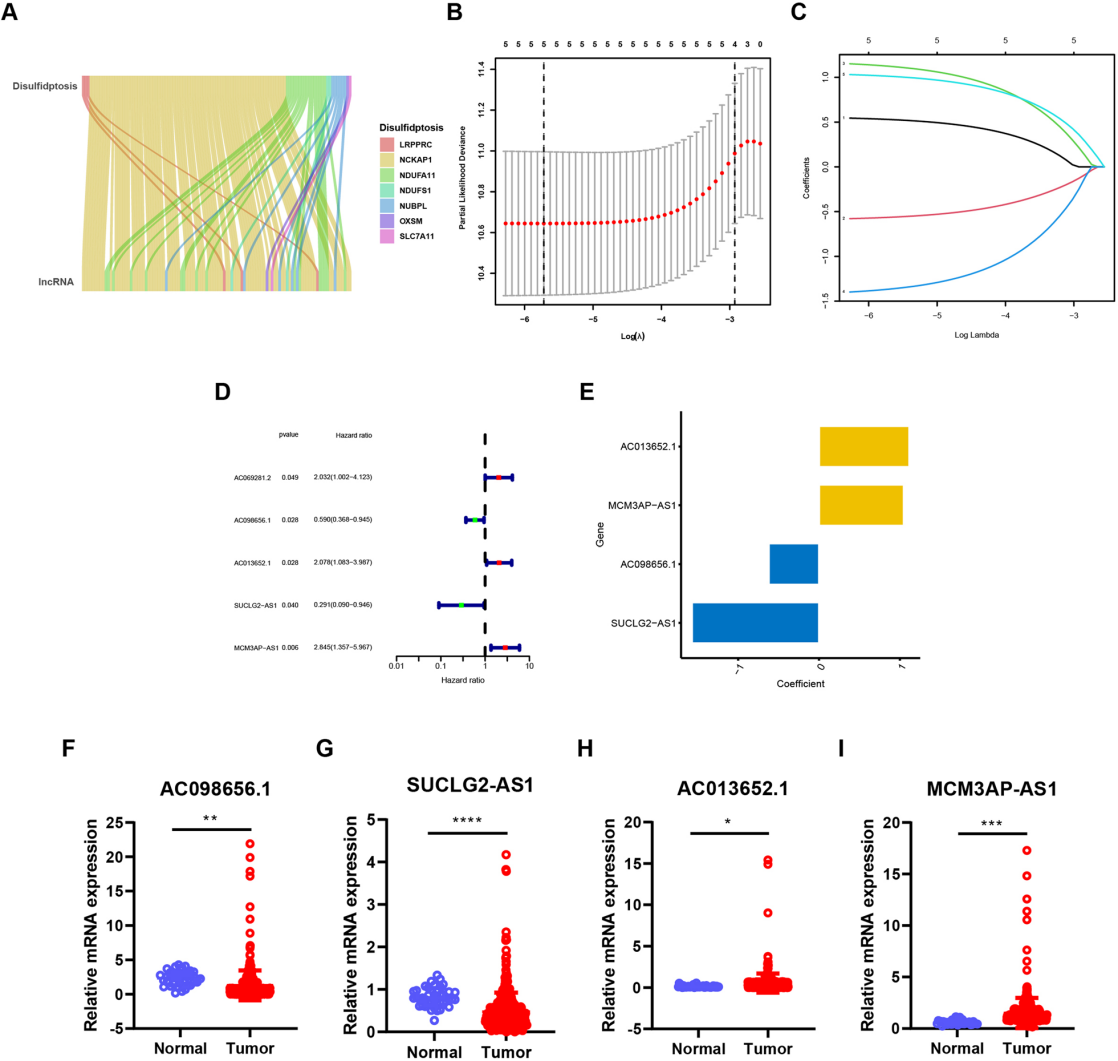
Identification of four disulfidptosis-related lncRNAs with prognostic value in the training group of COAD. **A** The Sankey diagram showing the relationship between disulfidptosis-related genes and lncRNAs. **B** and **C** LASSO expression was performed. **B** showing the cross-validation plot for the penalty term, and **C** showing plots for LASSO expression coefficients of the disulfidptosis-related lncRNAs. **D** Forest plot of disulfidptosis-related lncRNAs obtained by univariate Cox regression analysis. **E** The coefficient of four disulfidptosis-related lncRNAs. **F**–**I** Relative mRNA expression of the four disulfidptosis-related lncRNAs in normal colon and tumors. Samples of COAD were from TCGA. ****p < 0.0001; ***p < 0.001; **p < 0.01; *p < 0.05

We then performed data analysis to identify lncRNAs associated with disulfidptosis that could potentially regulate this process and serve as therapeutic targets in COAD. The expression profiles of ten genes reported to be linked to disulfidptosis were extracted from TCGA. Pearson’s correlation coefficient analysis (|Pearson R|> 0.4 and p < 0.001) was subsequently employed to identify approximately 100 disulfidptosis-related lncRNAs (Fig. 2A).

Next, we divided 448 COAD samples from the TCGA dataset into training and testing groups, ensuring no significant differences in clinicopathological features between the groups (Supplementary Table 2). Univariate Cox regression analysis was then performed on the training group to screen for lncRNAs with prognostic significance. Subsequently, LASSO regression analysis was employed to establish a risk signature based on the results of the univariate analysis (Fig. 2B–D). This analysis identified five lncRNAs (AC069281.2, AC098656.1, AC013652.1, SUCLG2-AS1, and MCM3AP-AS1) for further investigation. Multivariate Cox regression analysis was then used to identify lncRNAs that independently influenced overall patient survival. This analysis revealed four key lncRNAs: AC098656.1, AC013652.1, SUCLG2-AS1, and MCM3AP-AS1 (Fig. 2E). Notably, AC098656.1 and SUCLG2-AS1 emerged as protective factors, exhibiting higher expression in normal samples compared to tumor samples (Fig. 2F, G). Conversely, AC013652.1 and MCM3AP-AS1 acted as poor prognostic factors, showing higher expression in tumor samples (Fig. 2H, I).

### The risk signature was associated with the prognosis of patients with colon adenocarcinoma in the training group

Multivariate Cox regression analysis identified an optimal risk signature for prognostic prediction in COAD patients, based on the four identified disulfidptosis-related lncRNAs. The risk score for each sample was calculated using the following formula: Risk score = − 0.6217 × Exp (AC098656.1) − 1.5711 × E × p (SUCLG2-AS1) + 1.1163 × Exp (AC013652.1) + 1.0478 × Exp (MCM3AP-AS1). Using this formula, all samples were categorized into high- or low-risk groups based on the median risk score.

**Fig. 3:**
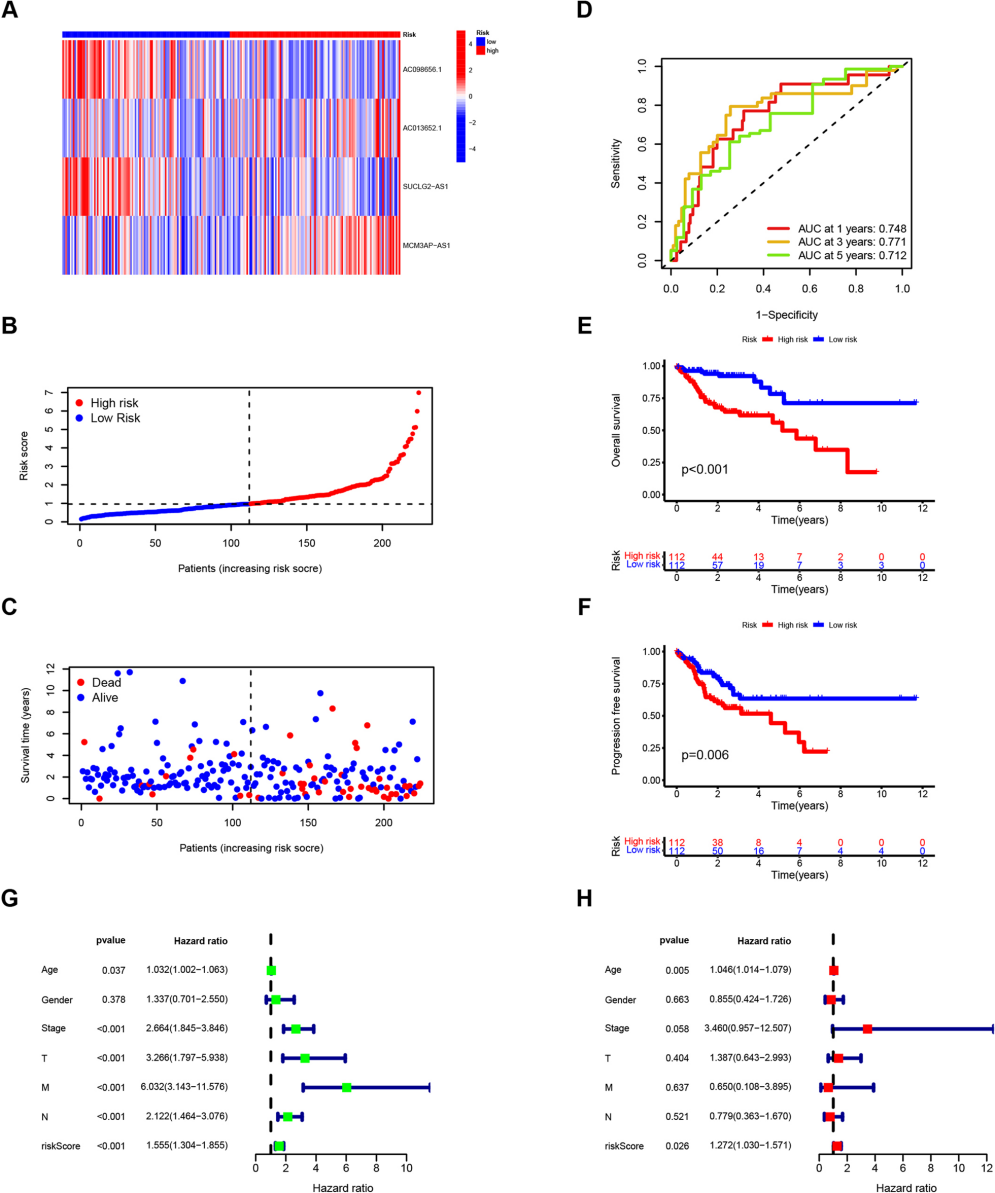
Identification of the prognostic value of the disulfidptosis-related lncRNAs in the training group of COAD patients. **A** Heatmap showing the expression of four lncRNAs in each tumor sample. **B**–**F** The risk score (**B**), survival time and status (**C**), ROC curve predicting the overall survival (**D**), Kaplan–Meier curve of overall survival (**E**) and progression-free survival (**F**) in low- and high-risk groups from COAD patients. **G** and **H** Univariate Cox regression analysis (**G**) and multivariate Cox regression analysis (**H**) of clinical characteristics and risk score in COAD samples in the training group. Samples of COAD were from TCGA

The distribution of the four lncRNAs across COAD patient tumor samples is depicted in a heatmap (Fig. 3A). Notably, as the risk score increased, the number of survivors decreased, while the number of deceased increased (Fig. 3B, C). To evaluate the prediction accuracy of the risk signature, we performed ROC analysis and used the area under the curve (AUC) as a metric. The AUCs were 0.748, 0.771, and 0.712 for one, three, and five years, respectively, indicating good prediction accuracy (Fig. 3D). Consistent with these findings, Kaplan–Meier survival curves revealed a significantly worse prognosis for patients in the high-risk group compared to the low-risk group, evident in both overall survival (p < 0.001) and progression-free survival (p = 0.006) (Fig. 3E, F). Furthermore, both univariate and multivariate Cox regression analyses demonstrated a significant correlation between the risk signature and patient survival. These results suggest that the risk signature can independently predict prognosis (Fig. 3G–H).

### Verification of the risk signature in the validation group and the entire cohort

**Fig. 4:**
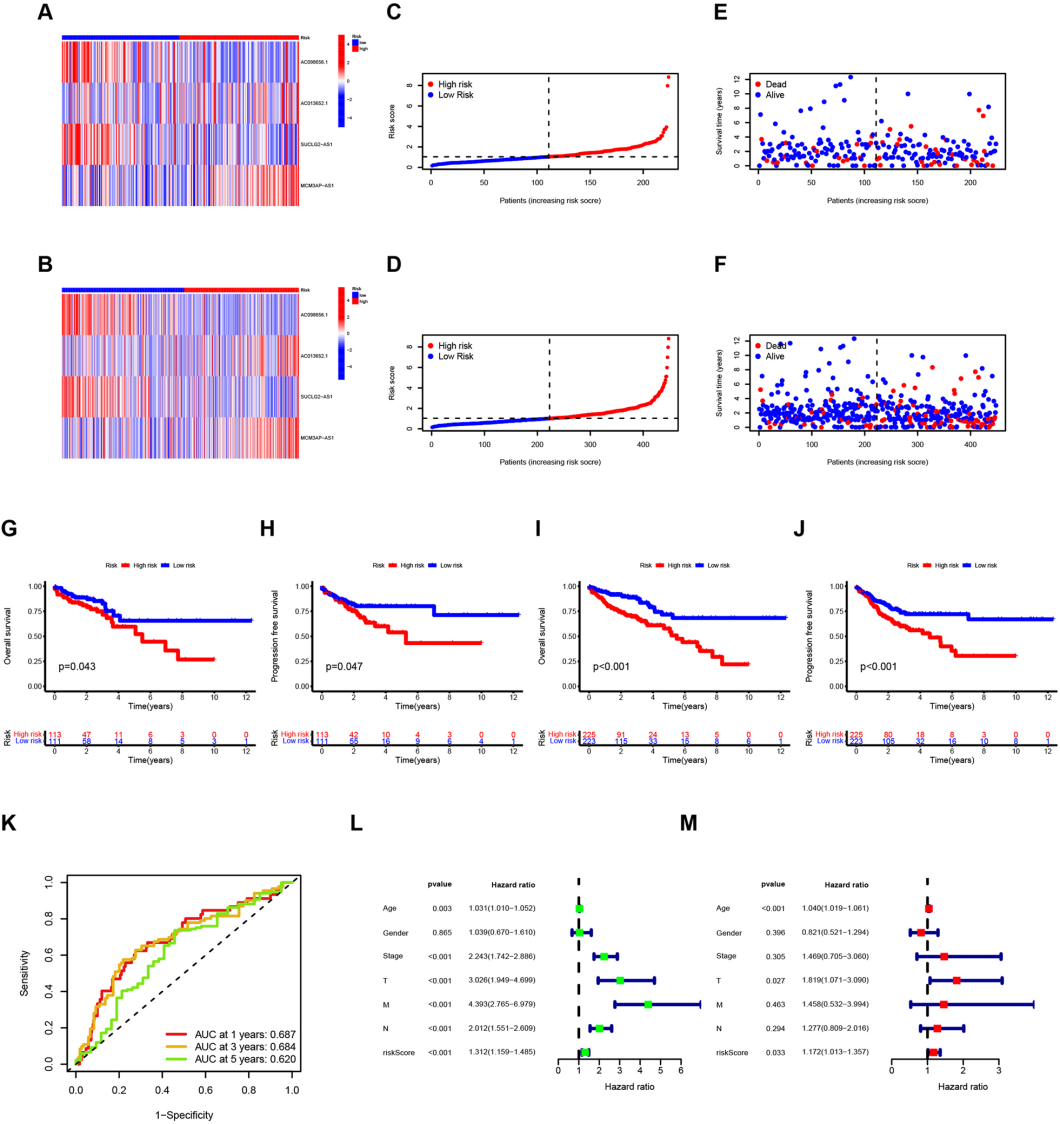
Identification of the prognostic value of the disulfidptosis-related lncRNAs in the testing group and entire cohort of COAD patients. **A**–**F** The expression of the four lncRNAs (**A**), distribution of low- and high-risk samples (**C**), and the correlation of risk score and survival time and status (**E**) in the testing group. The expression of four lncRNAs (**B**), distribution of low- and high-risk samples (**D**), and correlation of risk score and survival time and status (**F**) in the entire cohort. **G**–**J**. Kaplan–Meier curve of OS and PFS in testing group (**G** and **H**) and entire cohort (**I** and **J**). **K** ROC curve of the risk score in the entire cohort. **L** and **M** Univariate (**L**) and multivariate (**M**) Cox regression analyses of clinical characteristics and risk scores in entire cohort (**M**)

To validate the robustness of the aforementioned risk signature, we further evaluated its performance in both the testing group and the entire COAD cohort. As expected, the distributions of the four lncRNAs in the testing group and the entire cohort resembled those observed in the training group (Fig. 4A, B). Consistent with the training data, an increase in risk score was associated with a decrease in survival and a concurrent increase in mortality (Fig. 4C–F). Furthermore, Kaplan–Meier survival curves consistently demonstrated a worse prognosis for patients in the high-risk group compared to the low-risk group in both the testing group and the entire cohort (Fig. 4G–J). The AUCs for the entire cohort confirmed the excellent predictive power of the risk signature (Fig. 4K). Additionally, Cox regression analysis identified the risk signature as an independent prognostic indicator for COAD patients (Fig. 4L–M).

### The risk signature could predict better than other clinicopathological features

**Fig. 5:**
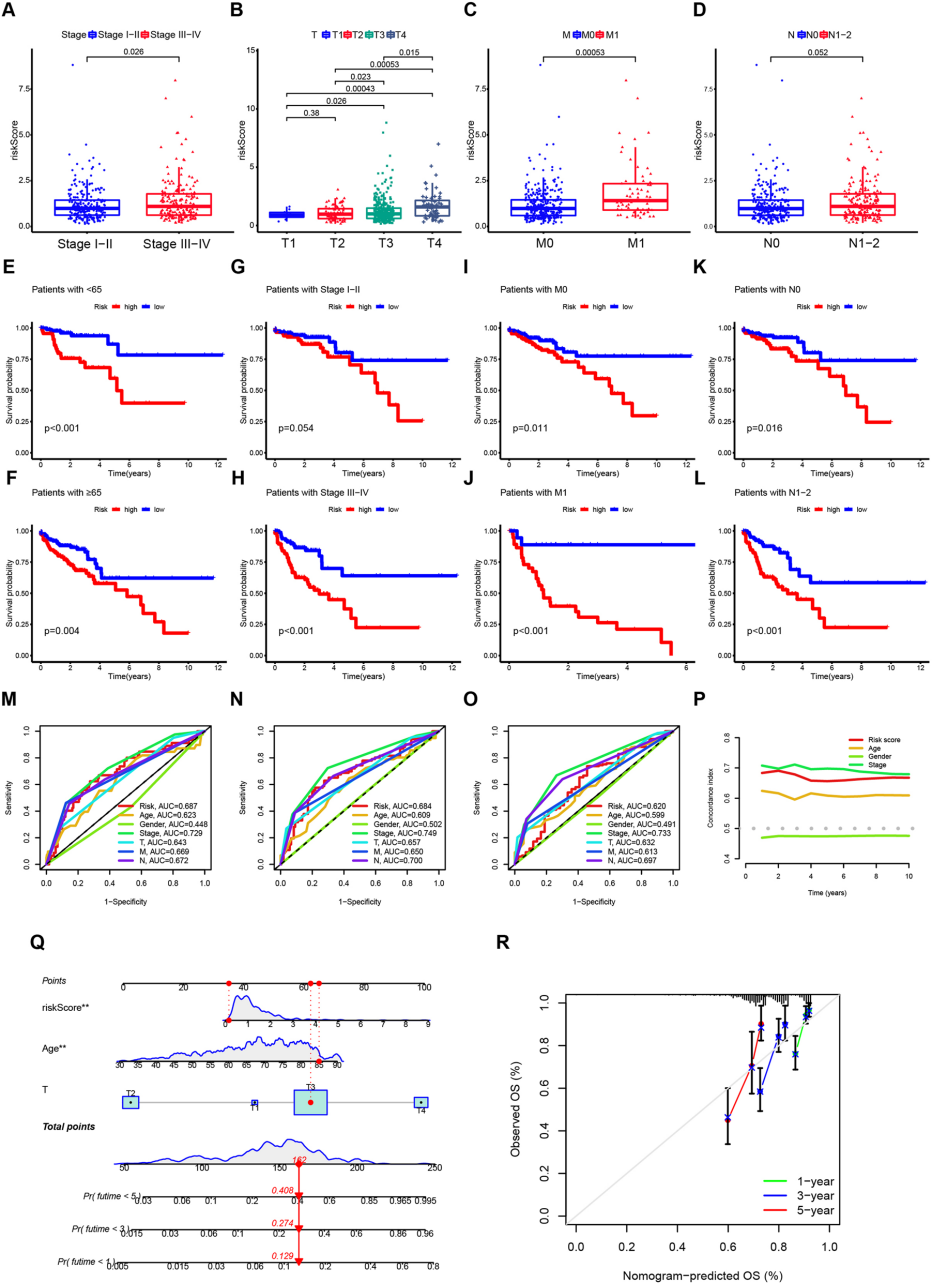
Relationship between risk score and clinicopathological characteristics. **A**–**D** Risk score in different stages of COAD patients. **E**–**L** Kaplan–Meier survival curves for patients aged < 65 or ≥ 65 years. Clinicopathological subgroups analysis of OS for COAD patients. Stage 1–2 (**F**), M0 (**G**), N0 (**H**), Stage 3–4 (**J**), M1 (**K**), N1-2 (**L**). **M**–**O** ROC curve of 1-year (**M**), 3-year (**N**), and 5-year (**O**) OS for the risk signature and other clinical characteristics. P. The concordance index (c-index) for the risk signature and other clinical characteristics of COAD patients. **Q** and **R** The nomogram (**Q**) and the calibration curves (**R**) established by the risk score and other clinical characteristics. ****p < 0.0001; ***p < 0.001; **p < 0.01; *p < 0.05

First, we analyzed the correlation between the risk score and other clinicopathological features. Patients with higher risk scores exhibited a trend towards more advanced tumor stage (TNM classification) (Fig. 5A–D). To further investigate the risk signature’s predictive power in different patient subgroups, we performed subgroup survival analysis. The analysis revealed a significantly worse prognosis for samples with higher risk scores, regardless of age (Fig. 5E, F) or pathological status (Fig. 5H–L). Notably, even though no significant difference was observed in stage I-II patients, the high-risk group still displayed a poorer prognosis (Fig. 5G). Next, we compared the prognostic ability of the risk signature with other clinicopathological features using ROC survival curves (Fig. 5M–O). The risk score achieved AUCs of 0.687 at one year, 0.684 at three years, and 0.620 at five years, indicating good accuracy. Consistent with these findings, the c-index analysis confirmed the strong prognostic value of the risk signature compared to other factors (Fig. 5P). A nomogram was constructed to provide a visual and quantitative tool for predicting 1-, 3-, and 5-year survival rates (Fig. 5Q). To assess the nomogram’s accuracy, we employed a calibration curve. The results demonstrated a high degree of concordance between the predicted and actual survival rates (Fig. 5R).

### Functional analysis of DEGs between the high- and low- risk groups

**Fig. 6:**
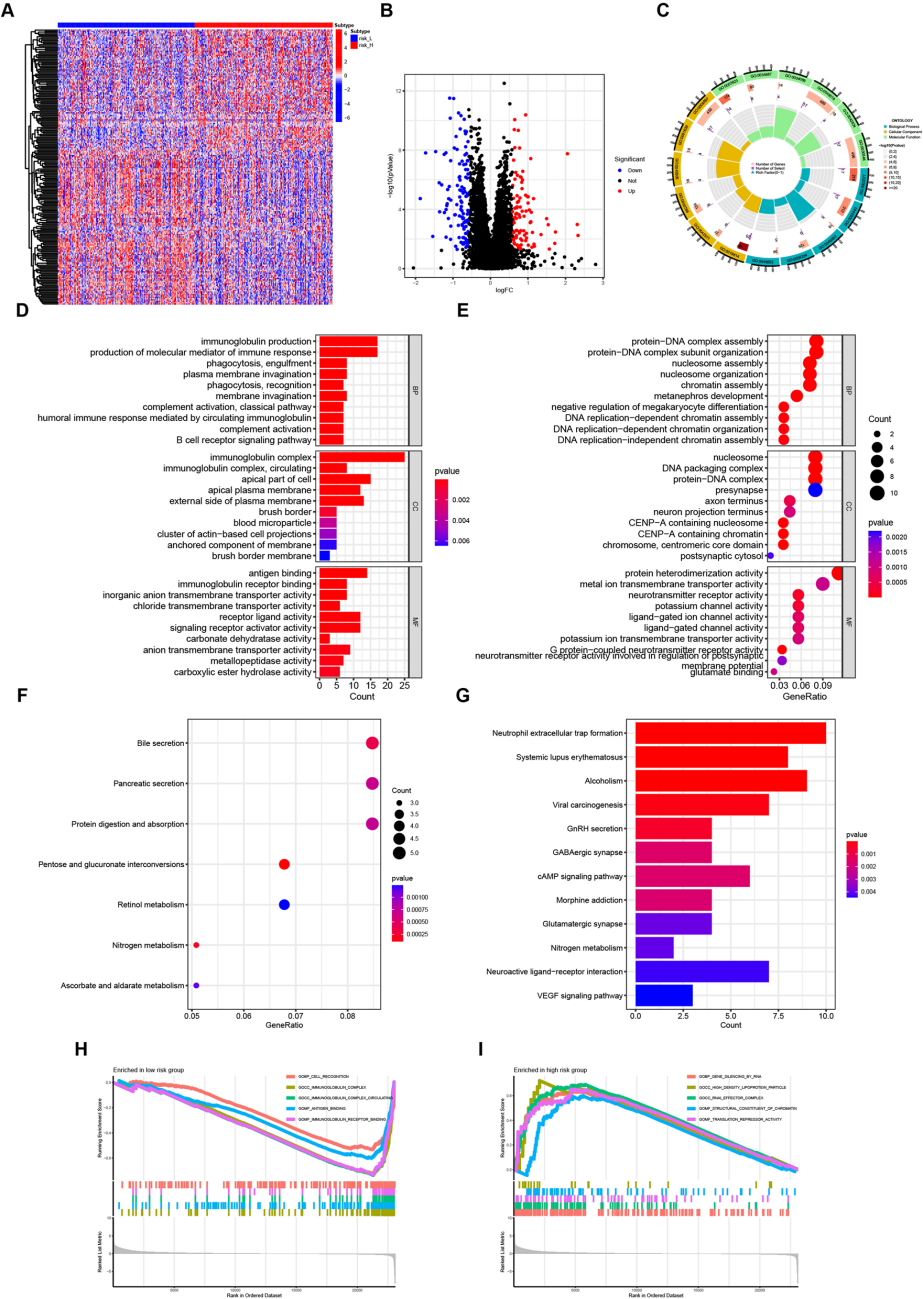
Enrichment analyses of differentially expressed genes between high-risk and low-risk groups. **A** and **B** Heatmap (**A**) and volcano plot (**B**) showing differential expressed genes between low-risk and high-risk groups. |FoldChange|> 1.5, adj P < 0.05. **C**–**E** GO analyses of differentially expressed genes in the two groups (**C**), highly expressed genes in the low-risk group (**D**), and high-risk group (**E**) **E** and **F** KEGG analyses of highly expressed genes in the low-risk group (**F**), and the high-risk group (**G**)** H** and **I** Gene Set Enrichment Analysis (GSEA) for interpreting gene expression data in the low-risk group (**H**), and the high-risk group (**I**)

We next investigated the function of DEGs between the high- and low-risk groups. Differential expression analysis and enrichment analysis were performed to identify these DEGs. DEGs were identified using a threshold of |FoldChange|> 1.5 and adjusted p-value < 0.05, as visualized in the heatmap and volcano plot (Fig. 6A, B). GO enrichment analysis revealed that genes upregulated in the low-risk samples were significantly enriched in immune-associated biological functions (Fig. 6C, D). These functions included immunoglobulin production, complement activation, and the B cell receptor signaling pathway in biological processes (BP), immunoglobulin complex in cellular components (CC), and antigen binding and immunoglobulin receptor binding in molecular functions (MF). These findings suggest a more active immune response in the low-risk group. In contrast, the high-risk group exhibited enrichment in DNA replication-associated signaling pathways (Fig. 6E). KEGG pathway analysis further indicated enrichment of Protein digestion and absorption, and Bile secretion pathways in the low-risk group (Fig. 6F), while Neutrophil extracellular trap formation, cAMP signaling pathway, and VEGF signaling pathway were enriched in the high-risk group (Fig. 6G). GSEA confirmed these observations, demonstrating enrichment of abundant immune-related functions in the low-risk samples, while high-density lipoprotein particle and RNAi effector complex pathways were enriched in the high-risk samples (Fig. 6H, I).

**Fig. 7:**
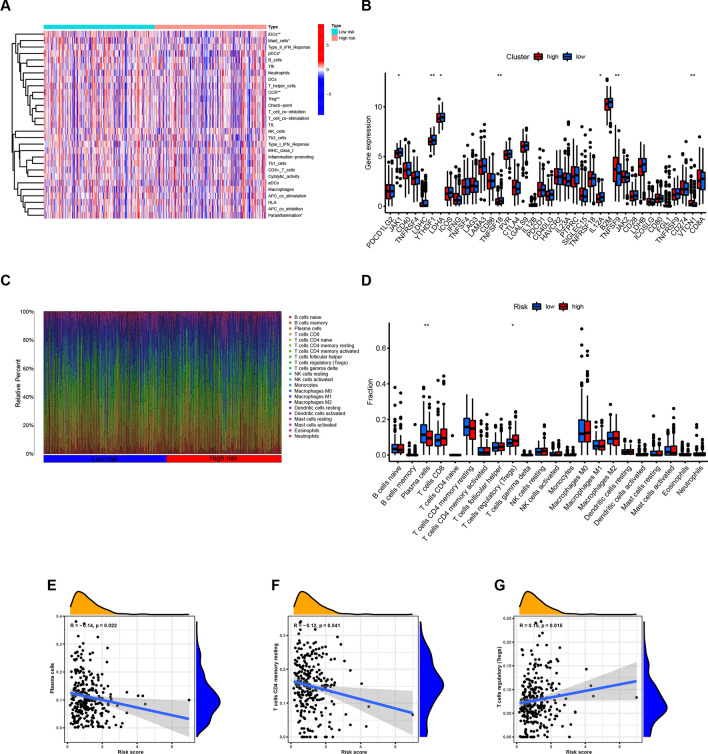
The difference of tumor immune microenvironment between high- and low-risk groups in COAD patients. **A** and **B** The heatmap and bar chart revealed the expression of immune cells in the two groups. **C** and **D** The distinction between infiltrating immune cells as calculated by CIBERSORT algorithm. **E**–**G** The correlation between the risk score and infiltrating immune cells calculated by CIBERSORT algorithm in TCGA. ****p < 0.0001; ***p < 0.001; **p < 0.01; *p < 0.05

Based on the enrichment analysis above, the risk score emerged as a strong indicator of immune responses in COAD patients. We further investigated the impact of the risk signature on immune responses within the tumor microenvironment. First, we employed ssGSEA to analyze the potential correlation between the risk score and immune-associated functions. Significant differences between the two groups were observed in immune cell populations including immature dendritic cells (iDCs), mast cells, regulatory T cells (Tregs), and pro-inflammatory cells (Fig. 7A). We then explored the relationship between the risk score and immune checkpoint genes. The results showed that the expression of JAK1, YTHDF1, and TNFSF18 increased with higher risk scores, while TNFSF9 and VTCN1 expression exhibited the opposite trend (Fig. 7B). Furthermore, the CIBERSORT algorithm was used to quantify the differences in immune cell proportions between the two groups (Fig. 7C, D). The analysis revealed a strong correlation between the risk score and the proportion of immune cells. As the risk score increased, the fractions of plasma cells (p = 0.022) and CD4 memory resting T cells (p = 0.041) decreased, while Tregs showed an increase (p = 0.015) (Fig. 7E–G). These findings are consistent with the previous observation that samples with lower risk scores had higher immune scores and displayed a more active immune response. In conclusion, these results provide novel insights into the immune microenvironment of COAD tumors.

## Knockdown of AC013652.1 or MCM3AP-AS1 increased disulfidptosis under glucose starvation conditions in COAD cells

**Fig. 8:**
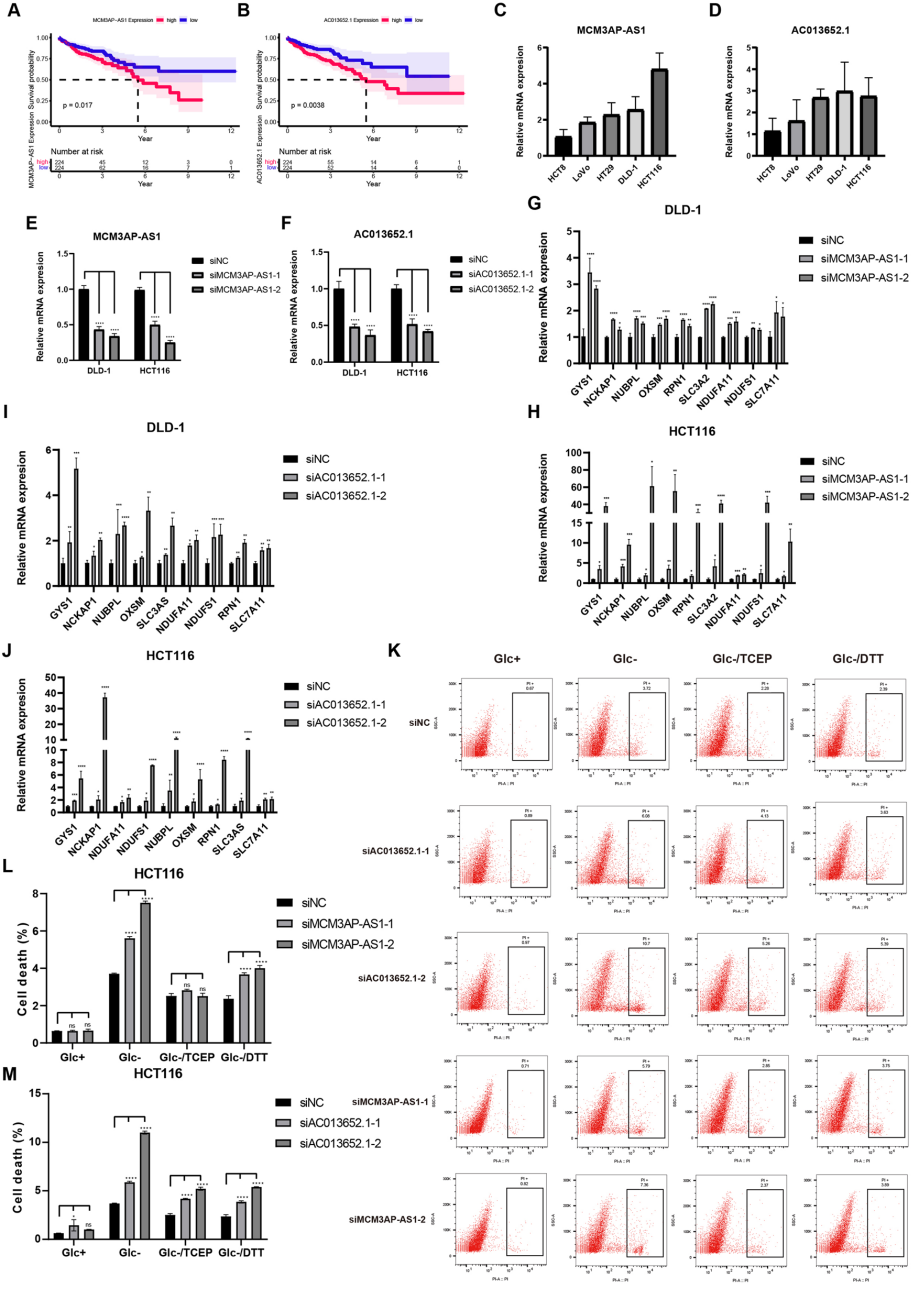
The roles of AC013652.1 and MCM3AP-AS1 in regulating disulfidptosis in COAD. **A** and **B** Kaplan–Meier curve of OS for patients with different expression levels of MCM3AP-AS1 (**A**), and AC013652.1 (**B**). **C** and **D** The expression of MCM3AP-AS1 (**C**) and AC013652.1 (**D**) in COAD cells confirmed by qRT-PCR. **G**–**J** The expression of disulfidptosis associated genes after knocking down the MCM3AP-AS1 (**G**, **H**), or AC013652.1 (**I**, **J**) under glucose starvation conditions. **K**–**M** The effect of MCM3AP-AS1 or AC013652.1 knockdown on the death of HCT116 after cultured in glucose-free medium for 12 h and that treated with 0.5 mM DTT or 1 mM TCEP. The statistical analyses were conducted by two-way ANOVA. ****p < 0.0001; ***p < 0.001; **p < 0.01; *p < 0.05

Among the four disulfidptosis-related lncRNAs, high expression of AC013652.1 or MCM3AP-AS1 correlated with worse patient prognosis (Fig. 8A, B). To validate the functional role of these lncRNAs in COAD cells in vitro, we investigated the expression levels of AC013652.1 and MCM3AP-AS1 (Fig. 8C, D). DLD-1 and HCT116 cells were selected for further studies, and qRT-PCR analysis confirmed successful knockdown of the lncRNAs (Fig. 8E, F). After knocking down the expression of two lncRNAs, the expression of disulfidptosis associated genes were all up-regulated under both glucose and glucose starvation conditions (Fig. 8G–J, Supplementary Fig. 2). Furthermore, under glucose starvation conditions, knockdown of AC013652.1 or MCM3AP-AS1 significantly increased the percentage of PI-positive dead cells. Notably, this cell death was partially rescued by DTT and TCEP treatment (Fig. 8K–M, Supplementary Fig. 3). These findings suggest that AC013652.1 and MCM3AP-AS1 act as poor prognostic indicators and may regulate disulfidptosis in COAD cells in vitro*.*

## Discussion

Disulfidptosis is a recently discovered form of regulated cell death linked to cellular metabolism. However, its involvement and regulation in both physiological and pathological processes remain poorly understood. In this study, we aimed to elucidate the prognostic value of disulfidptosis-associated genes in COAD patients by analyzing data from TCGA. Leveraging the molecular profiles of COAD patients from TCGA, we identified a four-lncRNA signature associated with disulfidptosis in COAD.

The lncRNA signature could not only serve as an independent risk factor but also predict the immune infiltration within the tumor microenvironment. Disulfidptosis elucidates how cells can initiate cell death in response to cystine imbalance during glucose deprivation. Imbalance of intracellular cystine and cysteine can lead to significant changes in the tumor immune microenvironment. The depletion of cysteine sensitizes the prostate cancer cells to agents that promote immune checkpoint inhibition [29]. Moreover, deprivation of cystine impairs the function of tumor-infiltrating CD8 + T cells [30]. The deficiency of cystine-glutamate antiporter preserves antitumor immunity [31]. Besides, the cells are experiencing oxidative stress due to the significant consumption of NADPH in order to alleviate intracellular disulfide stress. Cellular oxidative stress can lead to the production of inflammatory cytokines and chemokines [32]. All these factors could contribute to variations in tumor immune microenvironment between the high- and low-risk groups.

Subsequent in vitro experiments using human COAD cell lines confirmed the occurrence of disulfidptosis under glucose starvation conditions. Furthermore, these experiments demonstrated that AC013652.1 and MCM3AP-AS1 inhibit disulfidptosis. LncRNAs have emerged as promising targets for cancer diagnosis, treatment, and prognosis, including in COAD [33–35]. Previous studies have reported the tumor-promoting roles of lncRNAs AC013652.1 and MCM3AP-AS1 in various cancers. Interestingly, AC013652.1 was identified as one of fifteen ferroptosis-associated lncRNAs that are closely linked to poor prognosis in COAD [36]. Additionally, research suggests that AC013652.1 can serve as an effective prognostic predictor and guide immunotherapies and personalized treatment for gastric cancer patients [37]. MCM3AP-AS1, another essential cancer-related lncRNA, has been identified as part of a metabolism-related lncRNA signature that can predict outcomes in COAD patients, potentially aiding in personalized prevention and treatment strategies [38].

Solute Carrier Family 7 Member 11 (SLC7A11) encodes a component of a transport system that exhibits high specificity for cysteine and glutamate. It can increase cell uptake of cystine and reduce cystine to cysteine. Therefore, disulfidptosis is more likely to occur in cells with high expression of SLC7A11. In this study, we demonstrated that AC013652.1 and MCM3AP-AS1 regulate disulfidptosis. Interestingly, silencing either lncRNA led to an increase in SLC7A11 expression. LncRNAs can exert their effects through various mechanisms, including directly binding to transcriptional regulatory genes or influencing post-transcriptional modifications and protein stability, such as p53 [39, 40]. However, the precise molecular mechanisms by which AC013652.1 and MCM3AP-AS1 regulate SLC7A11 and other disulfidptosis-associated genes remain to be elucidated. Furthermore, it is unclear whether the effects of these lncRNAs on disulfidptosis solely rely on SLC7A11 expression or involve additional targets. These questions warrant further investigation.

Liu et al. previously reported a high enrichment of actin cytoskeletal molecules in the disulfidptosis-modified fraction [41]. Consistent with these findings, we observed changes in the actin cytoskeleton of COAD cells under starvation conditions. These changes manifested as abnormal cell morphology, with actin retractions around the cell edges and a collapse of lamellipodia. Notably, the alterations in actin observed in our COAD cell model suggest a potentially universal role for actin in disulfidptosis. Indeed, the actin cytoskeleton is a well-established target of programmed cell death in both plants and animals. Studies have documented an active and functional role for the cytoskeleton in regulating actin dynamics during PCD. For instance, disruption of actin filament integrity has been shown to induce apoptosis in human airway epithelial cells [42]. However, the precise mechanisms by which cells succumb to cytoskeletal collapse during disulfidptosis and the factors leading to these actin cytoskeleton changes remain unclear and warrant further investigation.

Our study is the first report about the involvement of disulfidptosis in COAD, which may bring new breakthroughs and advancements to the field of COAD treatment. By inducing disulfide stress within tumor cells, disulfidptosis can be triggered to inhibit the growth and metastasis of tumors. Therefore, disulfidptosis can be used as a combined treatment strategy in conjunction with other therapies to enhance treatment effectiveness. Besides, we demonstrated the regulation of disulfidptosis by AC013652.1 or MCM3AP-AS1. These two lncRNAs can also serve as therapeutic targets to promote cancer disulfidptosis, thereby improving the prognosis of COAD.

Our study has some limitations that warrant acknowledgment. First, the data analysis relied on reported disulfidptosis-associated genes, whose specificity for disulfidptosis requires further validation. Second, our study heavily relied on public database data, which may introduce biases compared to real-world scenarios. Third, in vivo experiments are necessary to verify the regulation of disulfidptosis by AC013652.1 or MCM3AP-AS1 in COAD models. In conclusion, further basic research is needed to refine the findings of this study and comprehensively elucidate the role of disulfidptosis in COAD development, particularly its regulation by lncRNAs.

## Conclusions

In this study, we demonstrated the presence of disulfidptosis in COAD cell lines. We further explored the prognostic value of disulfidptosis-associated genes in COAD patients. Additionally, we established a risk signature based on disulfidptosis-related lncRNAs, which emerged as a promising prognostic indicator. Finally, loss-of-function assays confirmed the roles of AC013652.1 and MCM3AP-AS1 in regulating disulfidptosis.

### Supplementary Information

Supplementary Material 1.

## Data Availability

The data are available within the Article, Supplementary Information, or available from the authors upon request. Source data are provided with this paper.
