# Supplementary material for: Establishment of disulfidptosis-related LncRNA signature as biomarkers in colon adenocarcinoma
Source: Cancer Cell Int. 2024 May 27;24:183. doi: 10.1186/s12935-024-03374-6 (PMC11131243; doi:10.1186/s12935-024-03374-6)
Supplement: Supplementary file 1 — Supplementary Material 1. [file 12935_2024_3374_MOESM1_ESM.docx]

**Supplementary Figures**


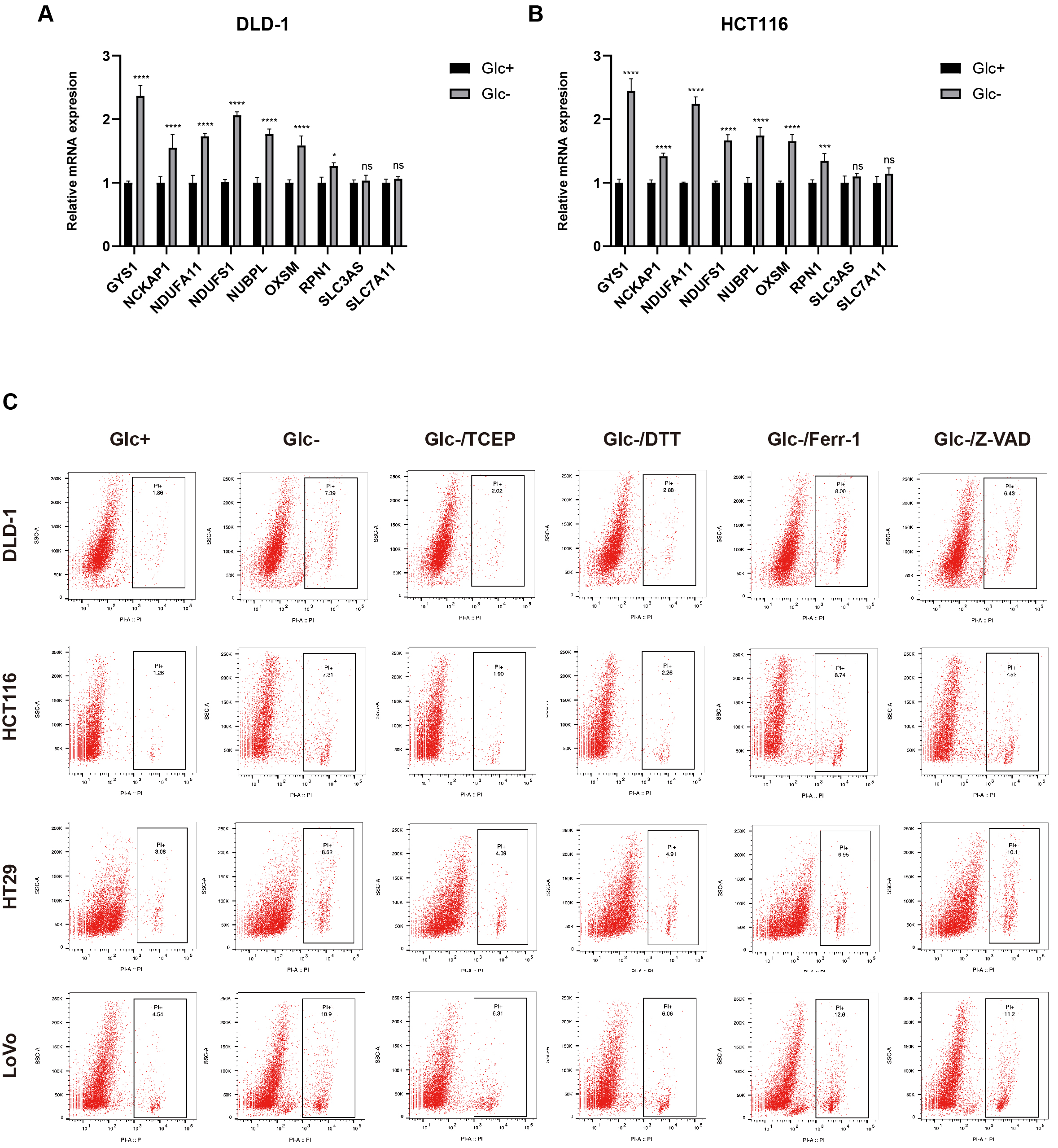


**Supplementary Figure 1**. **The involvement of disulfidptosis in colon cancer.**

A-B. The expression of disulfidptosis associated genes after glucose starvation for 12 hours in DLD-1 (A) and HCT116 (B). The statistical analyses were done by two-way ANOVA. C. The percentage of PI positive death cells after glucose starvation for 12 hours, and that treated with 0.5mM DTT, 1 mM TCEP, 10μm Ferr-1 or 10μm Z-VAD showed by flowcytometry. ****p < 0.0001; ***p < 0.001; **p < 0.01; *p < 0.05.


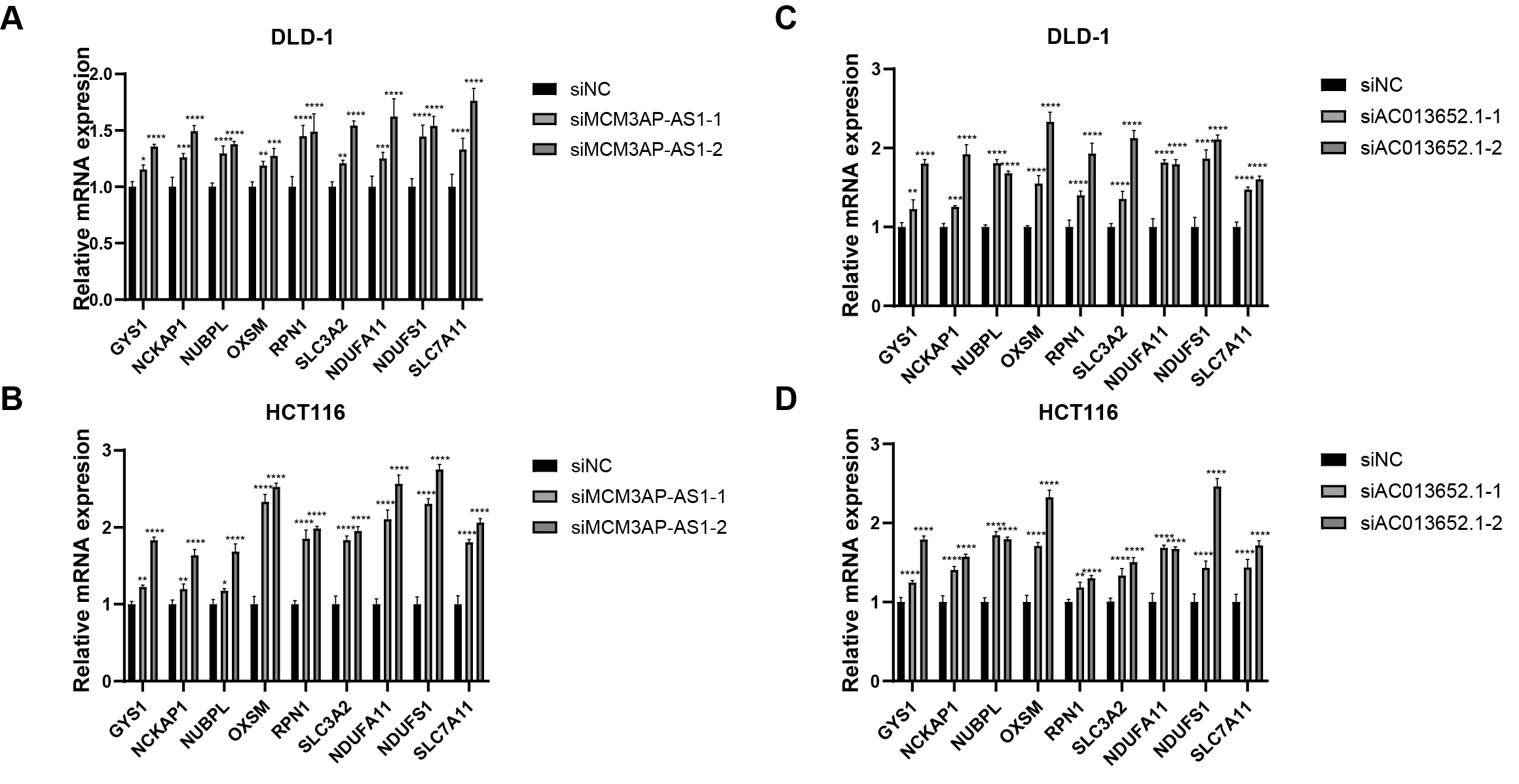


**Supplementary Figure 2**. **Knockdown of AC013652.1 or MCM3AP-AS1 upregulated** **disulfidptosis associated genes expression under conditions of glucose presence.**

A-D. The expression of disulfidptosis associated genes after knocking down the MCM3AP-AS1 (A-B), or AC013652.1 (C-D) under conditions of glucose presence. The statistical analyses were done by two-way ANOVA. ****p < 0.0001; ***p < 0.001; **p < 0.01; *p < 0.05.


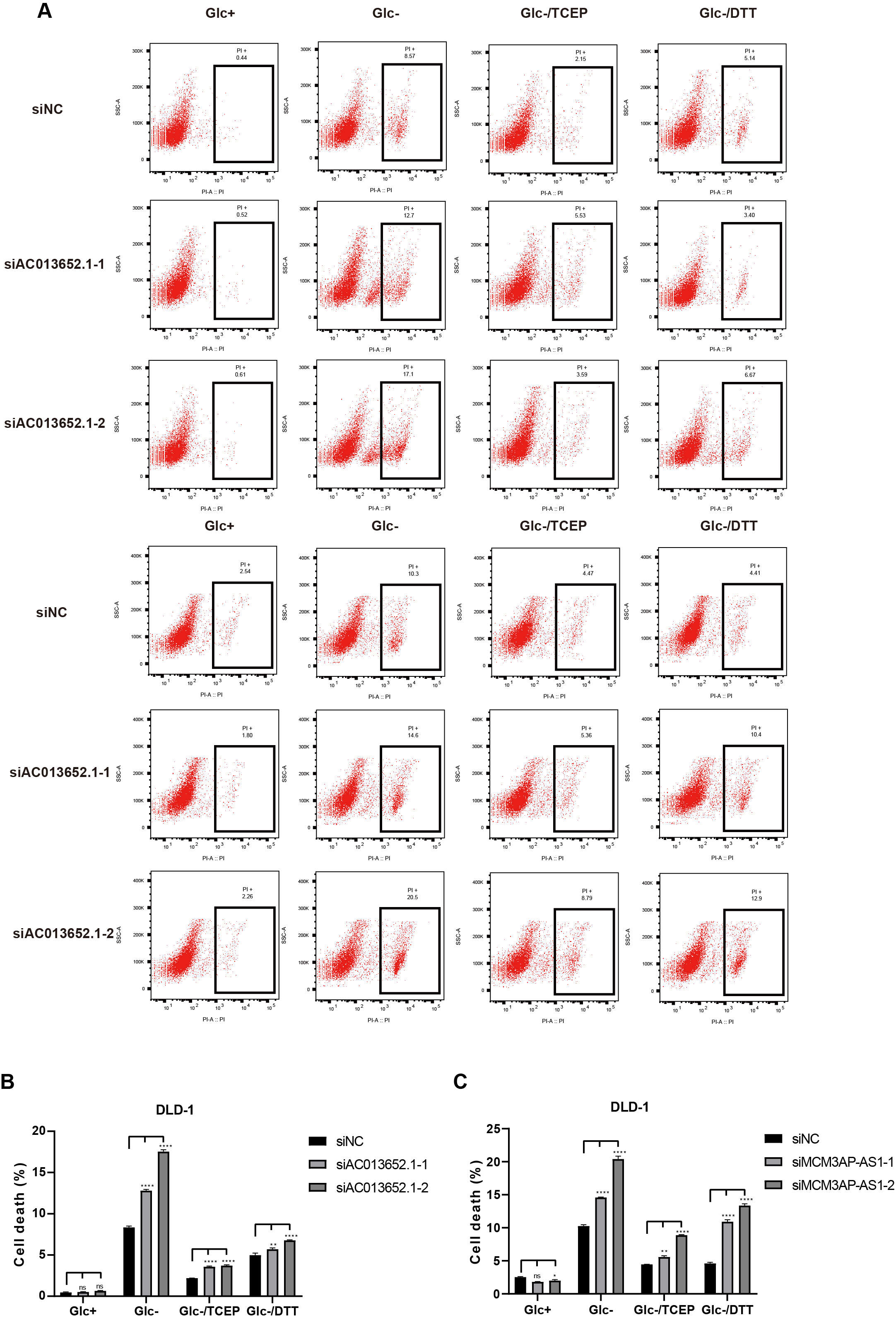


**Supplementary Figure 3.** **Knockdown of AC013652.1 or MCM3AP-AS1 increased disulfidptosis under glucose starvation conditions in COAD cells**

A-C The effect of MCM3AP-AS1 or AC013652.1 knockdown on the death of DLD-1 after cultured in glucose-free medium for 12h and that treated with 0.5mM DTT or 1 mM TCEP. The statistical analyses were done by two-way ANOVA. ****p < 0.0001; ***p < 0.001; **p < 0.01; *p < 0.05.

**Supplementary Table**

**Table 1 Summary of disulfidptosis associated genes**

| Disulfidptosis associated genes |
| --- |
| GYS1 NDUFS1 OXSM LRPPRC NDUFA11  NUBPL NCKAP1 RPN1 SLC3A2 SLC7A11 |

**Table 2 Clinicopathological comparison of COAD patients in the training group and the testing group**

| Covariates | Type | Total | Train | Test | P-value |
| --- | --- | --- | --- | --- | --- |
| Age | <=65  >65 | 184(41.07%)  264(58.93%) | 82(36.61%)  142(63.39%) | 102(45.54%)  122(54.46%) | 0.0681 |
| Gender | FEMALE  MALE | 214(47.77%)  234(52.23%) | 109(48.66%)  115(51.34%) | 105(46.88%)  119(53.12%) | 0.7766 |
| Stage | Stage I  Stage II  Stage III  Stage IV  unknow | 75(16.74%)  176(39.29%)  124(27.68%)  62(13.84%)  11(2.46%) | 39(17.41%)  89(39.73%)  67(29.91%)  27(12.05%)  2(0.89%) | 36(16.07%)  87(38.84%)  57(25.45%)  35(15.62%)  9(4.02%) | 0.5999 |
| T | T1  T2  T3  T4  unknow | 10(2.23%)  76(16.96%)  305(68.08%)  56(12.5%)  1(0.22%) | 5(2.23%)  38(16.96%)  155(69.2%)  26(11.61%)  0(0%) | 5(2.23%)  38(16.96%)  150(66.96%)  30(13.39%)  1(0.45%) | 0.9473 |
| M | M0  M1  MX  unknow | 330(73.66%)  62(13.84%)  49(10.94%)  7(1.56%) | 174(77.68%)  27(12.05%)  19(8.48%)  4(1.79%) | 156(69.64%)  35(15.62%)  30(13.39%)  3(1.34%) | 0.1064 |
| N | N0  N1  N2 | 266(59.38%)  102(22.77%)  80(17.86%) | 135(60.27%)  47(20.98%)  42(18.75%) | 131(58.48%)  55(24.55%)  38(16.96%) | 0.6416 |

**Table 3 The primers used in this study**

| Genes | primer |
| --- | --- |
| GAPDH forward  GAPDH reverse | 5′-CAGGAGGCATTGCTGATGAT-3′  5′-GAAGGCTGGGGCTCATTT-3′ |
| MCM3AP-AS1 forward  MCM3AP-AS1 reverse | 5′- GCTGCTAATGGCAACACTGA-3′  5′- AGGTGCTGTCTGGTGGAGAT-3′ |
| AC013652.1 forward  AC013652.1 reverse  GYS1 forward  GYS1 reverse | 5′- CTCAAAGCATCCTGGGGGTC-3′  5′- GGGTCATCCTACACCCAACC-3′  5′- GCGCTCACGTCTTCACTACTG-3′  5′- TCCAGATGCCCATAAAAATGGC-3′ |
| NDUFS1 forward  NDUFS1 reverse | 5′- TTAGCAAATCACCCATTGGACTG-3′  5′- CCCCTCTAAAAATCGGCTCCTA-3′ |
| OXSM forward  OXSM reverse  NDUFA11 forward  NDUFA11 reverse  NUBPL forward  NUBPL reverse  NCKAP1 forward  NCKAP1 reverse  RPN1 forward  RPN1 reverse  SLC3A2 forward  SLC3A2 reverse  SLC7A11 forward  SLC7A11 reverse | 5′- CAATATCCAGATTGCATAGGCGA-3′  5′- CGATCCCAAACCAGGTGAGTT-3′  5′- GCCGAAGGTTTTTCGTCAGTA-3′  5′- GGAGGATTGAGTGTGACTCTGT -3′  5′- CTGAGATGTTTCGCAGAGTCC-3′  5′- CAAGGGTCTGTGCTAGTTTCC-3′  5′- TCCTAAATACTGACGCTACAGCA-3′  5′- GCCTCCTTGCATTCTCTTATGTC-3′  5′- GGCCAAGATTTCAGTCATTGTGG-3′  5′- CTTCGTTGGATAGGGAGAGTAGA-3′  5′- TGAATGAGTTAGAGCCCGAGA-3′  5′- GTCTTCCGCCACCTTGATCTT-3′  5′- TCTCCAAAGGAGGTTACCTGC-3′  5′- AGACTCCCCTCAGTAAAGTGAC-3′ |
